# Di-(2-ethylhexyl) Phthalate-Induced Hippocampus-Derived Neural Stem Cells Proliferation

**DOI:** 10.22074/cellj.2016.4862

**Published:** 2016-12-21

**Authors:** Alireza Abdanipour, Ali Noori-Zadeh, Seyed Alireza Mesbah-Namin, Salar Bakhtiyari, Reza Nejatbakhsh, Iraj Jafari Anarkooli

**Affiliations:** 1 Department of Anatomy, School of Medicine, Zanjan University of Medical Sciences, Zanjan, Iran; 2 Department of Clinical Biochemistry, Faculty of Medical Sciences, Tarbiat Modares University, Tehran, Iran; 3 Student Research Committee Center, Ilam University of Medical Sciences, Ilam, Iran

**Keywords:** Proliferation, *Sox2*, Neural Stem Cells

## Abstract

The brain and spinal cord have a limited capacity for self-repair under damaged conditions. One of the best options to overcome these limitations involves the use of phytochemicals as potential therapeutic agents. In this study, we have aimed to investigate the effects of di-(2-ethylhexyl) phthalate (DEHP) on hippocampus-derived neural stem cells (NSCs) proliferation to search phytochemical candidates for possible treatment of neurological diseases using endogenous capacity. In this experimental study, neonatal rat hippocampus-derived NSCs were cultured and treated with various concentrations of DEHP (0, 100, 200, 400 and 600 µM) and *Cirsium vulgare (C. vulgare)* hydroethanolic extract (0, 200, 400, 600, 800 and 1000 µg/ml) for 48 hours under *in vitro* conditions. Cell proliferation rates and quantitative *Sox2* gene expression were evaluated using MTT assay and real-time reverse transcription polymerase chain reaction (RT-PCR). We observed the highest average growth rate in the 400 µM DEHP and 800 µg/ml *C. vulgare* extract treated groups. *Sox2* expression in the DEHP-treated NSCs significantly increased compared to the control group. Gas chromatography/mass spectrometry (GC/ MS) results demonstrated that the active ingredients that naturally occurred in the *C. vulgare* hydroethanolic extract were 2-ethyl-1-hexanamine, n-heptacosane, 1-cyclopentanecarboxylic acid, 1-heptadecanamine, 2,6-octadien-1-ol,2,6,10,14,18,22-tetracosahexaene, and DEHP. DEHP profoundly stimulated NSCs proliferation through *Sox2* gene overexpression. These results provide and opportunity for further use of the C. vulgure phytochemicals for prevention and/or treatment of neurological diseases via phytochemical mediated-proliferation of endogenous adult NSCs.

In the neuroscience field, there is an ongoing, increasing tendency to further research applications of molecules derived from nature to treat brain abnormalities and associated-psychiatric problems. Traditional Chinese medicine uses different types of thistles [species: *Cirsium vulgare (C. vulgare)*] to prepare decoctions that alleviate inflammation, seizures, and disorders of the central nervous system (CNS) (1-4). In the adult brain, neurogenesis persists throughout life and neural stem cells (NSCs) (5) primarily reside in the dentate subgranular zone (6), rostral subventricular zone, and other brain regions (7). It is believed that neurogenesis increases in response to brain injuries, such as stroke (8) as well as with neurodegenerative diseases such as Alzheimer’s (9), Huntington’s (10), and multiple sclerosis (11, 12). On the other hand, it has been accepted that endogenous NSCs may to some extent replace damaged neural cells by self-repair (13). 

The newly generated cells can migrate into the damaged regions and differentiate into functional neural (14) as well as glial cells (15). However, the capacity of CNS self-repair is obviously not enough to treat or cure neurodegenerative diseases. It is well known that neurotrophic factors have invaluable potential in the treatment of CNS diseases and traumatic injuries through promoting endogenous NSCs proliferation and neuron formation. However, the CNS is a site protected by various barriers; a significant challenge to neurotrophic therapy is the difficulty associated with the delivery of hydrophilic proteins to the damaged brain (16). One option to overcome these limitations is the use of biochemical molecules known as phytochemicals as therapeutic agents. The discovery of novel phytochemicals that affect NSCs can pave the way to stimulate the proliferative response involving the endogenous capacity of NSCs. In this study, we have aimed to investigate the effects of di-(2-ethylhexyl) phthalate (DEHP) on hippocampus-derived NSCs proliferation. *Sox2* gene expression, as a main NSC self-renewal promoting factor, was assessed by immunocytochemistry and quantitative real-time reverse transcription polymerase chain reaction (RT-PCR). The results of this research showed that phytochemical mediated-proliferation stimulation of the endogenous adult NSCs could be a tremendous opportunity for future treatment of neurological diseases.

All experimental procedures and protocols used in this project were reviewed and approved by the Ethics Committee for the use of experimental animals at Tarbiat Modares University.

In this experimental study, after deep anesthesia, 3-day-old neonatal Sprague-Dawley rats were used to isolate NSCs. The hippocampus was separated, and then mechanically crushed. Acutase (Invitrogen, UK) and collagenase (Invitrogen, UK) were used for enzymatic digestion purposes at 37˚C for 30 minutes after which fetal bovine serum (FBS, Gibco, USA) was added to neutralize the enzymes. The suspension was filtered through a 70 µm nylon mesh and centrifuged at 400 g for 10 minutes. The obtained cells were cultured in DMEM/F12 medium (Invitrogen, UK) that contained basic fibroblast growth factor (bFGF, Invitrogen, UK), epidermal growth factor (EGF, Invitrogen, UK), 2% B27 (Gibco, USA), 1% penicillin-streptomycin (Gibco, USA), and 3% FBS at temperature of 37˚C and in 5% CO_2_. After 24 hours, the medium was changed. After reaching 70-80% confluency, the cells were passaged using trypsin (0.05%) and EDTA (0.02%) at 1 ml per 25 cm_2_ of the surface area. Passage-3 NSCs were cultured on cover slides and fixed with 3% paraformaldehyde for 20 minutes at room temperature (RT), followed by a permeabilization step with 0.3 % Triton X-100 for 30 minutes at RT. For immunostaining, cells were incubated with mouse anti-Nestin monoclonal and anti-Sox2 antibodies (Abcam, UK) followed by incubation with FITC-conjugated rabbit antimouse secondary antibody (Millipore, UK). Nuclei were counterstained with ethidium bromide. The cells were visualized and photographed using a fluorescence inverted microscope (Olympus, Japan).

In this experimental study, *C. vulgare* hydroethanolic extract was prepared using the Soxhlet method (17). The flowers of *C. vulgare* (Herbarium No.: 13268) were collected from the Estil wetland (Astara) of the Gilan Province of Iran. The collected flowers were dried in the shade and subsequently ground. The resultant shadedried powder (100 g) was subjected to extraction in a Soxhlet extractor with 70% ethanol (hydroethanolic) for 12 hours (extract yield: 13%), and *C. vulgare* extract was collected. The extract was placed in glass containers in the oven for 24 hours at 50˚C. The remaining solvent was kept at 4˚C. Gas chromatography/mass spectrometry (GC/ MS) analysis of *C. vulgare* hydroethanolic extract was performed using GC-MSD Agilent GC, a gas chromatography interfaced to a mass spectrometer equipped with an HP5 od 0.25 µm×30 m column. We used an electron ionization system with an ionizing energy of 70 eV for GC/MS detection. Pure helium gas was the carrier gas at a constant flow rate (1 ml/minute) and a 1 µl injection volume (split ratio: 1:20) with an injector temperature of 250˚C and ion-source temperature of 280˚C. The oven temperature was programmed from 110˚C (isothermal for 2 minutes) with an increase rate of 10˚C/minute to 200˚C, followed by 5˚C/minute to 280˚C, and finally a 10 minute isothermal at 280˚C. Mass spectra were taken at 70 eV. Total GC/MS running time was 46 minutes. The relative percent amount of each component was measured by comparing its average peak value to the total areas. Interpretation of the mass spectrum of GC/MS was done using Wiley7n.L libraries. We compared the resultant mass spectrum from the unknown composition of this work to the spectrum of the known components stored in this library. 

Passage-3 NSCs were treated with 0 (control group), 200, 400, 600, 800 and 1000 µg/ml *C. vulgare* hydroethanolic extract in 96-well plates for 48 hours. For verification purposes, five replicates were considered for each concentration of hydroethanolic *C. vulgare* extract. NSCs in the other experimental groups received 0 (control group), 100, 200, 400, and 600 µM DEHP for 48 hours in 96-well plates. Cell proliferation rates were evaluated using the MTT assay and *Sox2* gene expression.

We used the MTT assay to evaluate cellular proliferation. MTT stock solution (5 mg/ml) was added to each assayed culture to equal one-tenth the original culture volume. The culture was allowed to incubate for 4 hours according to the manufacturer’s instructions (Sigma-Aldrich, Germany). After 48 hours of treatment we replaced the medium with 20 µl of a freshly prepared solution of MTT. The supernatant of the cells was removed after 4 hours of incubation and formazan crystals dissolved in 100 µl Dimethyl sulfoxide (DMSO, Sigma, Germany) at RT for a few minutes. Subsequently, the absorbance was measured at 570 nm. The relative cell viability as a percentage was calculated as follows: A570 of treated samples/A570 of untreated samples×100 (18, 19).

Total RNA was extracted using a Pure Link RNA Mini Kit (Invitrogen, UK) according to the manufacturer’s instructions. In both groups, purified RNA (DNA-free) was used to synthesize 20 μl cDNA with the Revert aid™ First Strand cDNA Synthesis Kit (Fermentas, Germany) according to the manufacturer’s instructions. The prepared cDNAs were used in real-time RT-PCR to quantify *Sox2* gene expression fold changes. In these reactions, *β2m* gene was the internal control. The PCR reactions were prepared at a 20 μl final volume using SYBR Green PCR Master Mix (Applied Biosystems) and carried out for 40 cycles (Applied Biosystems cycler). We used the Pfaffl formula to analyze relative changes in *Sox2* gene expression (20). Table 1 lists the primers used in this experiment.

After a few hours, we identified self-renewing neural-like cells that had multipolar processes and growth cone-like features. After several days we observed small spheroids (Fig.1A). We cultured the intact neurospheres without dissociation in six-well adherent plates coated with poly-L- lysine. The cultured cells formed rosette-like structures (Fig.1B). Progenitor cell markers *Nestin* and *Sox2* were used to calculate the purity of passage-3 NSCs. Hippocampus-derived NSCs from spheroids showed strong positivity for *Nestin* (99.7 ± 0.3) and *Sox2* (98.4 ± 0.83, Fig.1C-F).

MTT assay results showed increased proliferation potency in treated NSCs compared with the control groups (Fig.2A, B). NSCs treated with 800 µg/ ml *C. vulgare* hydroethanolic extract (151.54 ± 6.2%) and 400 µM DEHP (174.1 ± 10.58%) had the highest, statistically significant average growth rate compared to the control groups (P<0.05).

Figure 2C shows the quantitative real-time RT- PCR *Sox2* gene expression pattern results for NSCs treated with the 0 and 400 µM concentrations of DEHP. *Sox2* mRNA in the DEHP-treated NSCs (1.27 ± 0.02) significantly increased compared with the untreated control group (0.76 ± 0.01).

The components present in the *C. vulgare* hydroethanolic extract were identified by GC/ MS analyses. The active principles with their retention time, molecular concentration (percent) in the hydroethanolic extract, and CAS number are presented in Table 2.

**Table 1:** Primer sequences and gene amplicon sizes accessed by real-time reverse transcription polymerase chain reaction (RT-PCR)


Gene	Accession no.	Sequence (5ˊ→ 3ˊ)	Size(bp)

*Sox2*	NM_001109181.1	F: CTCTCCCCTTCTCCAGTTC	223
		R: GTTACCTCTTCCTCCCACT	
*β2m*	NM_012512	F: CCCAACTTCCTCAACTGCTACG	243
		R: TTACATGTCTCGGTCCCAGGTG	


**Fig.1:**
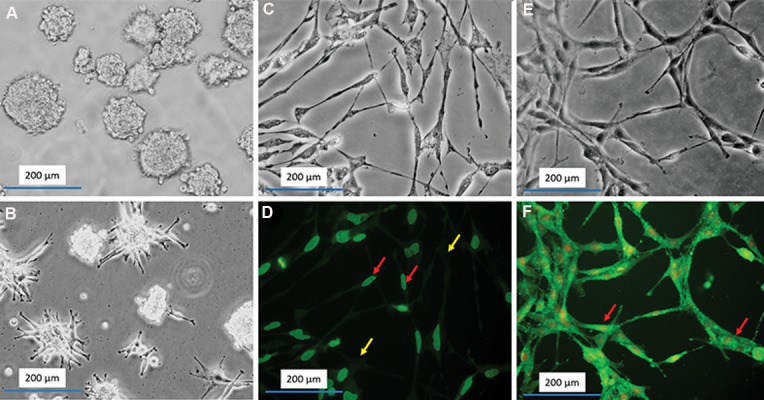
Isolation and culture of hippocampus-derived neural stem cells (NSCs). A. Phase contrast micrographs of neurospheres in suspen- sion culture, B. Represents rosette-like structures after 7 days of culture, C, D. Phase contrast and Immunocytochemistry Nestin positive NSCs, respectively, E, F. Phase contrast and Immunocytochemistry Sox2 positive NSCs, respectively. The Nestin and Sox2 proteins marker is green (FITC-conjugated secondary antibody) and the red nuclei are (counterstaining with ethidium bromide). Red and yellow arrows indicate to positive and negative cells, respectively (magnification: ×200).

**Fig.2:**
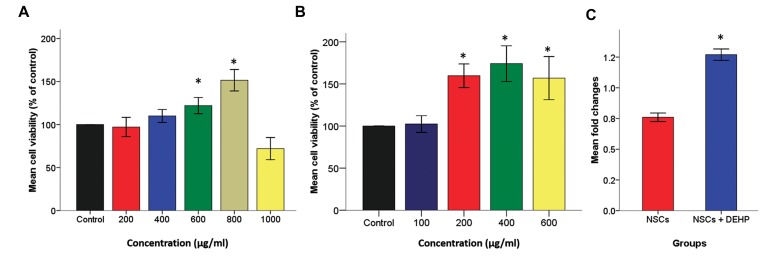
Proliferative effects of *Cirsium vulgare (C. vulgare)* extracts and di-(2-ethylhexyl) phthalate (DEHP) treatment on neural stem cell (NSC) viability. A. Dose-response hippocampus-derived NSC viability at different concentrations of *C. vulgare* extract detected by the MTT assay, B. Dose-response hippocampus-derived NSC viability at DEHP detected by the MTT assay. Results are shown as mean percent viability relative to untreated cells, and C. Comparative bar of *Sox2* gene expression normalized with *β2m* gene expression in NSCs and 400 µM DEHP-treated NSCs for 48 hours. Each bar represents the average measurement of five replicates. Bar graphs indicate the mean ± SEM. *; P<0.05 significant compared to the control group.

**Table 2:** Components detected by gas chromatography/mass spectrometry (GC/MS) in the *Cirsium vulgare (C. vulgare)* hydroethanolic extract


Row	Compound	R	Percent	CAS number

1	2-Ethyl-1-hexanamine	25.718	13.96	000104-75-6
2	Di-(2-ethylhexyl) phthalate (DEHP)	35.541	33.53	000593-49-7
3	1-Cyclopentanecarboxylic acid	41.352	5.06	131534-41-3
4	1-Heptadecanamine	43.269	2.14	004200-95-7
5	2,6-Octadien-1-ol	44.141	6.64	000106-24-1
6	2,6,10,14,18,22-Tetracosahexaene	44.325	16.71	007683-64-9
7	n-Heptacosane	44.693	5.99	000117-81-7


We have shown that *C. vulgare* extract and DEHP increased NSCs proliferation according to the MTT assay and *Sox2* gene results with real-time RT-PCR. Neural cell death is accepted as a common feature of neurological diseases. A current approach to treat neurological diseases is the transplantation of neural cells with the intent to replenish damaged or dead cells (21, 22). Endogenous NSCs can compensate the shortages that arise with the treatment of many CNS diseases (23). On the other hand, using phytomedicine may also have crucial roles in the development of effective drugs to treat neurological diseases through endogenous capacities.

In this study, we have researched phytochemicals extracted from *C. vulgare* flowers as a source of DEHP in order to induce proliferation of hippocampus-derived NSCs. Phytochemicals contain anti-oxidative stress, anti-inflammatory, calcium antagonization, anti-apoptosis, and neurofunction regulation properties that exhibit preventive or therapeutic effects for various neurodegenerative diseases (24). Effective phytochemicals may be a safe alternative option that circumvents the shortcomings seen with cell implantation and neurotrophic factor therapy. They pave the way to achieve a realistic approach in the treatment of neurological diseases. We have demonstrated that *C. vulgare* extracted- micromolecules such as DEHP induce the proliferation rate of NSCs compared to the control group. Additionally, our data have shown that *Sox2* expression in the extract-treated cells significantly increased compared to the control group. DEHP is the most abundant phthalate in the environment. Akingbemi et al. (25) have reported that DEHP-induced LH overstimulation, in concert with Leydig cell hyperplasia, caused chronic elevations in serum T levels. Yamashita et al. (26) demonstrated that DEHP stimulated proliferative responses and cytokine productions of murine spleen cells in vitro.

In another study, Kang et al. (27) showed that DEHP-induced cell proliferation was involved in the inhibition of gap junctional intercellular communication and blocked apoptosis in mouse Sertoli cells. Studies showed that the proliferation of NSCs was under the control of two important genes, *Sox2* and *Nestin*. We have used Nestin protein expression as a marker to calculate the hippocampus-derived NSC purity. The Nestin protein is an intermediate filament protein expressed in dividing cells during the early stages of development in the CNS and peripheral nervous system. The Nestin protein is important for the proper survival and self-renewal of NSCs (28). Recent studies have revealed that the *Sox2* gene is an SRY-related transcription factor which encodes a high-mobility group DNA-binding motif and during development. *Sox2* is expressed in embryonic stem and neuroepithelial cells (29, 30). In the neurogenesis processes, the *Sox2* gene is expressed throughout the developing cells in the neural tube and also in the proliferating progenitors of the CNS. However, during the final cell cycle of progenitors and differentiation, *Sox2* gene is downregulated (31). *Sox2*-positive cells represent an undifferentiated, dividing cell population in the subgranular (32) zone of the adult dentate gyrus. In addition to self-renewal, *Sox2*-positive NSCs contribute to the production of differentiated cells; this phenomenon may be relevant to understanding how the self-renewal of NSCs is coupled with the generation of differentiated cells through *Sox2* gene expression (33). Other studies have shown that hippocampal development and NSCs maintenance require *Sox2*-dependent regulation of sonic hedgehog (34). In the current study, in order to determine the type of phytochemicals possibly involved in the stimulation of NSCs self-renewal, we analyzed *C. vulgare* extract by GC/MS. The data have shown that this extract contained aliphatic and aromatic compounds such as saturated and polyunsaturated fatty acids, aliphatic polyamine- like structures, alcohols, and acids. The role of polyamines in the promotion of the proliferation capacity of the cells is well documented (35). In conclusion, our results provide an invaluable opportunity for further investigations on the C. vulgure-extracted phytochemicals to prevent or treat neurological diseases in the future.
